# Surgical repair of a giant congenital right atrial aneurysm concomitant with Wolff‐Parkinson‐White syndrome: A case report and literature review

**DOI:** 10.1002/ccr3.5743

**Published:** 2022-04-18

**Authors:** Seyed Mohsen Mirhosseini, Mahmood Beheshti Monfared, Mohammad Khani, Sepideh Jafari Naeini

**Affiliations:** ^1^ Cardiovascular research center Shahid Beheshti University of medical sciences Tehran Iran; ^2^ Department of Cardiovascular Surgery Shahid Modarres Hospital Shahid Beheshti University of Medical Sciences Tehran Iran; ^3^ 556492 Shahid Modarres Hospital Shahid Beheshti University of Medical Sciences Tehran Iran

**Keywords:** cardiac surgical procedures, congenital, heart aneurysm, Wolff‐Parkinson‐White syndrome

## Abstract

Congenital right atrial aneurysms (RAA) have a wide range of clinical presentations and leads to various complications. Depending on the initial presentation and associated complications, a conservative or surgical approach may be considered. A patient suffering from a giant RAA associated with the Wolff‐Parkinson‐White syndrome, who underwent successful surgical treatment, is presented here.

## Graphical abstract

Congenital right atrial aneurysms have a wide spectrum of clinical presentations and maybe associated with cardiac arrhythmia, which necessitates complete evaluation before decision making.

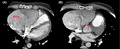

## 1 INTRODUCTION

Giant right atrial aneurysms (GRAA), which are also referred to as idiopathic enlargements of the right atrium (IERA), are very rare lesions with an unclear etiology and a broad spectrum of clinical presentations. They are mostly reported between the ages of 20 and 40 (1,2). Although the real prevalence has not been reported due to asymptomatic cases, a systematic review in 2021 analyzed 153 cases which had been reported until August 2019.^3^ It can manifest at any time from the antenatal period to old age.^3^ The Ebstein anomaly, pericardial effusion, pericardial cysts, and tumors should be considered as the differential diagnoses.^4^, ^5^

## 2 CASE REPORT

A 35‐year‐old man with a 20 years history of tachycardia, and palpitation was admitted to our center due to the deterioration of his symptoms. He had been administered 80 mg of sotalol on a daily basis. On admission, his pulse rate was 110–160 beats per minute, blood pressure was 96/68 mmHg, and his respiratory rate was 15/min; his body temperature was normal.

**FIGURE 1:**
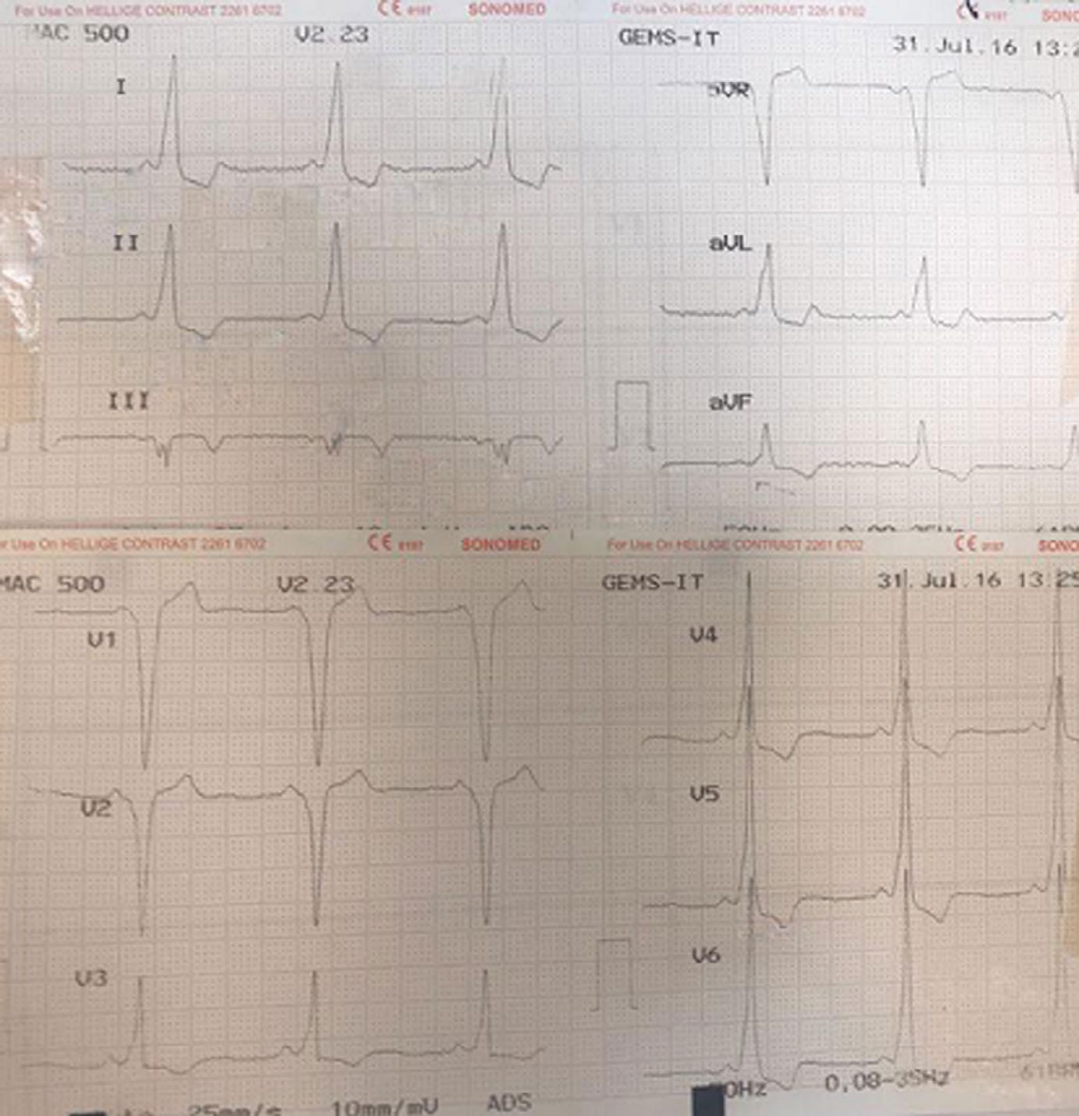
Electrocardiography revealed short PR interval and pre‐excitation in favor of right free wall accessory pathway

**FIGURE 2:**
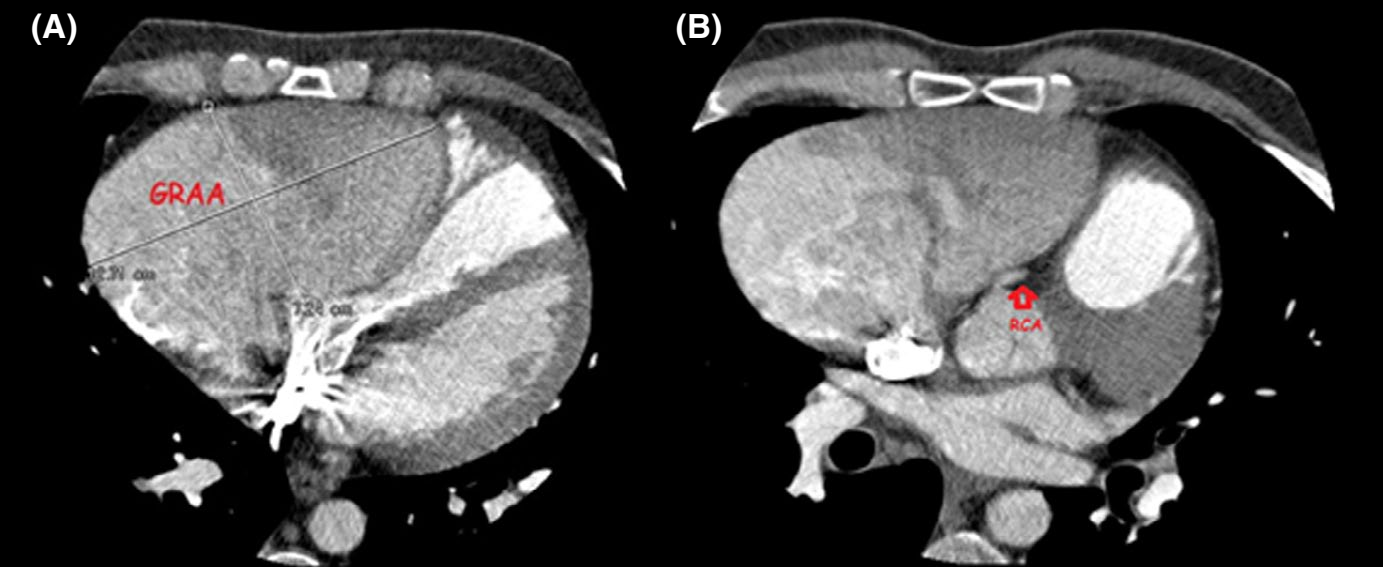
(A) (Left) Spiral CT‐ angiography was performed. A GRAA measuring approximately 127*122*72 mm is depicted in the anterior and right aspects of the cardiac chambers, just in front of the right atrium and right ventricle.^2^ (Right): Due to the compressive effect of the GRAA, the RCA and its branches and in particular, the SA‐ nodal branch, are displaced leftward.GRAA: Giant right atrial aneurysm, RCA: right coronary artery, SA node:Sinoatrial node

The initial electrocardiogram (ECG) revealed a short PR interval and delta wave, and the diagnosis of Wolff‐Parkinson‐White syndrome (WPW) was made based on the presence of palpitation and tachyarrhythmia (Figure 1). On admission, he was given a dose of sotalol (80 mg) and underwent direct current cardioversion. Due to the recurrent symptoms and hemodynamic concerns, electrophysiologic study (EPS) was conducted. A pre‐procedural chest X‐ray depicted an elevated cardiothoracic ratio as a result of huge right atrial dilation, and echocardiography demonstrated a normal left ventricle (LV) and preserved LV systolic function (Ejection fraction (EF): 50%), normal right ventricular (RV) size and severe RV systolic dysfunction with a huge right atrial aneurysm, which was compressing both ventricles. No significant valvular heart disease was reported except a mildly increased gradient across the RV inflow (tricuspid valve mean gradient: 3 mmHg and peak gradient: 8 mmHg) with localized pericardial effusion posterior to the LV (21 mm). Spiral computed tomography (CT) angiography of the thoracic aorta, pulmonary artery, and cardiac chambers was also performed (Video 1). A large blood‐containing structure measuring approximately 127*122 * 72 mm was depicted in the anterior and right aspects of the cardiac chambers, just in front of the right atrium and right ventricle (Figure 2A), without any thrombus formation. This structure had led to significant compression and displacement of the right atrium and right ventricle, including the tricuspid valve. The right coronary artery and its branches, particularly the SA (sinoatrial)—nodal branch, were displaced leftward and stretched considerably (Figure 2B). The upper level of this lesion was located above the level of bifurcation of the main pulmonary artery. The inferior vena cava (IVC) and superior vena cava (SVC) both drained normally into the right atrium. Because of adjacency to the right coronary artery and the broad muscular connection between the atrial aneurysm and the right ventricular free wall, a surgical approach was favored over electrophysiological study (EPS) ablation.

## 3 SURGICAL TECHNIQUE

**FIGURE 3:**
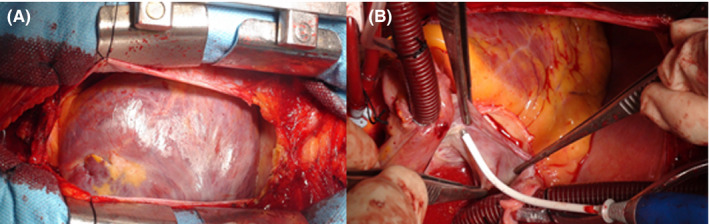
(A) (left): Giant right atrial aneurysm with a thin and translucent wall. (B) (right): Intraoperative radiofrequency ablation was performed for the accessory pathway

After cannulation through the right femoral artery and median sternotomy, a large aneurysm obstructing the whole operating field was observed (Figure 3A). The risk of aneurysmal wall rupture in the RA was very high due to the thin aneurysmal wall (Figure 3A). In order to decompress the heart and open the aneurysm safely, a mild hypothermic femoral bicaval cardiopulmonary bypass (CPB) was used. After a longitudinal incision, there was no evidence of thrombus formation or inflammation within the right atrial aneurysm, which was confirmed by simultaneous intraoperative transesophageal echocardiogram (TEE).

Intraoperative findings were a right coronary artery (RCA) protruding into the aneurysmal sac and normal atrial myocardium below the crista terminalis and right atrial appendage. After excision of the aneurysmal sac and affected right atrial wall, the defect was carefully repaired, avoiding trauma to the RCA. Radiofrequency ablation was used to ablate the right free wall accessory pathway (Figure 3B), without any complications.

## 4 OUTCOME AND FOLLOW‐UP

**FIGURE 4:**
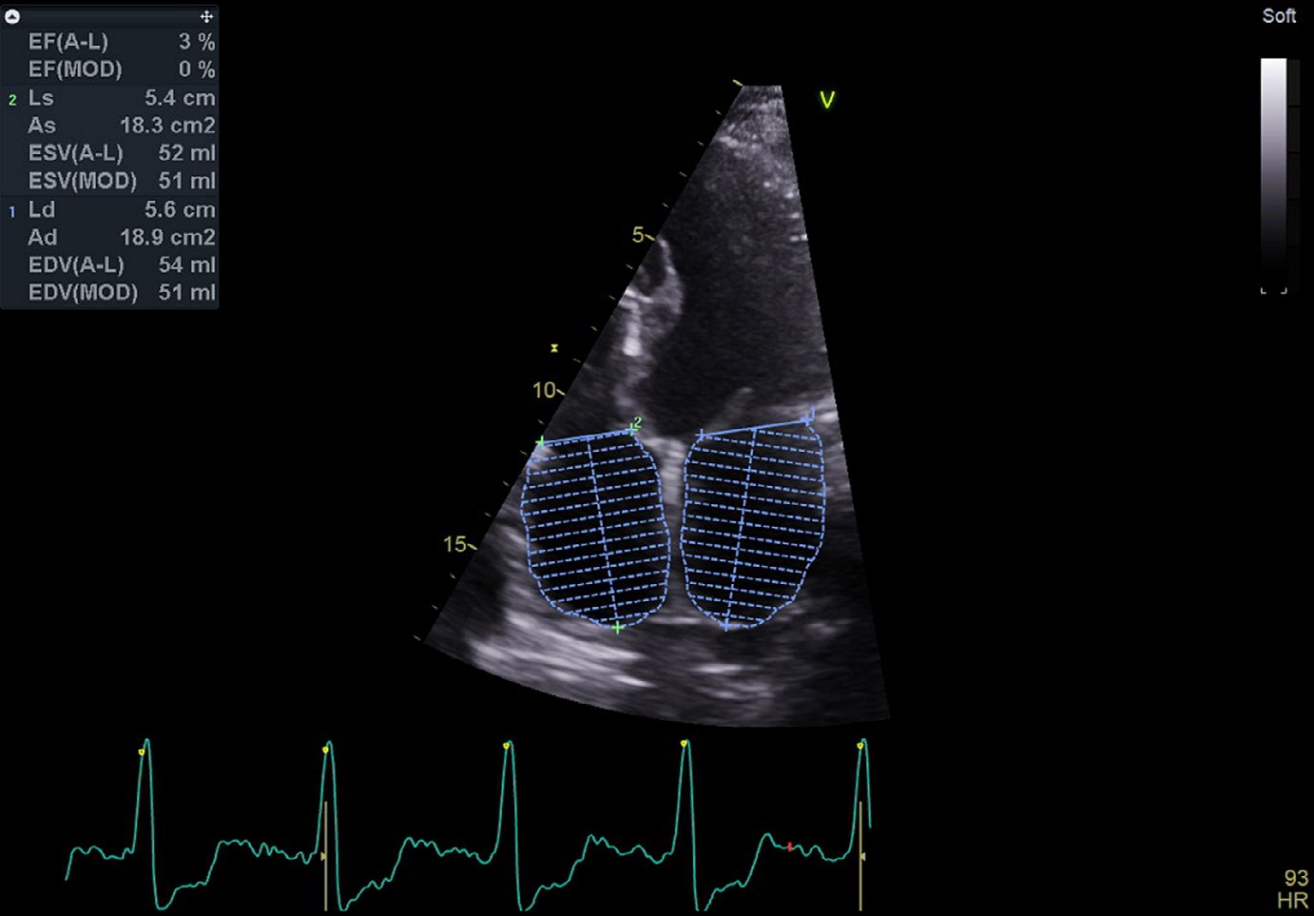
Transthoracic echocardiography after cardiac surgery: Residual mild RA enlargement

The patient was extubated 18 h after the surgery. Post‐operative recovery was uncomplicated, with no evidence of arrhythmia during the short term follow‐up, but the delta wave reappeared after several months on the surface ECG. Histological evaluation of the RAA wall revealed hypertrophied myocardium. Transthoracic echocardiography revealed mild enlargement of the RA with an LVEF of 55% with evidence of a small echodense ridge in the RV inflow with severe RV systolic dysfunction (in the early post‐operative period), which gradually improved over subsequent years. (Video 2, 3 from the 5 years follow‐up and Figure 4). On the 8th post‐operative day, the patient was discharged in a stable condition. Over an 18 months follow‐up period, he remained asymptomatic, but a recurrence of pre‐excitation was observed late in the follow‐up period, without any documented arrhythmias.

## 5 DISCUSSION

Atrial aneurysms can be differentiated from a diverticula based on the involvement of atrial layers. In contrast to diverticula, atrial aneurysms involve all the layers of the aneurysmal wall.^6^ Underlying mechanism may include defects in structural proteins, collagen or pectinate muscles, which lead to right atrial dilatation, regardless of right atrial pressure.^1^ A broad range of clinical presentations at various ages from the neonatal period to adulthood have been reported.^3^, ^7^ Resected tissue has been reported to show inflammatory cells but lack myofibrils based on one report.^8^ In our case, histological evaluation of the RAA wall showed hypertrophied myocardium and scattered fibrosis. While some cases are asymptomatic, a broad spectrum of symptoms, including supraventricular arrhythmias (even in the fetal period),^9^ thrombus formation, chest pain, fatigue, dyspnea, and syncope have been reported.^4^, ^7^, ^10^, ^11^, ^12^ In our case, the patient presented with palpitation due to WPW, which is very rarely associated with a GRAA and only a few cases have been reported in the literature.^13^, ^14^ Some experts have recommended using anticoagulants to prevent thrombus formation and administering anti‐arrhythmic medications to prevent arrhythmic complications.^15^ The underlying mechanism of arrhythmia in these patients is not fully understood, but according to studies that have reported the recurrence of sinus rhythm after resection of RAAs, atrial dilatation, myocardial fiber disorientation, and conduction system abnormalities have been proposed as possible mechanisms.^16^ Severe and refractory arrhythmia has also been reported in the presence of normal atrial tissue in histological assessment.^17^ In our case, although the patient regained normal sinus rhythm after surgical ablation, it did not last for a long time and the follow‐up ECG demonstrated recurrence of the delta wave with fewer episodes of tachycardia reported by the patient. In spite of the presence of tricuspid annular dilation and tricuspid valve regurgitation in some reports, which necessitate ring annuloplasty,^18^ our patient did not show significant regurgitation.

The optimal treatment of GRAA is yet to be determined. A conservative approach using low‐dose aspirin and regular follow‐up has been considered in asymptomatic patients with mild‐to‐moderate atrial dilatation. Surgery is preserved for atrial arrhythmia, clot formation, severe atrial dilatation, and the compressive effects of the aneurysm in patients with a low risk of mortality.^4^, ^17^, ^19^ The majority of studies favor CPB during aneurysmectomy, while a report by Joshi et al has suggested performing off‐pump aneurysmectomy using fine vascular clamps.^4^, ^10^ The use of CPB is based on the presence of a thin aneurysmal wall and significant risk of rupture, particularly during nonsurgical ablative intervention for the arrhythmia. Moreover, the unexpected finding of a protruding RCA makes an open heart procedure more favorable. Radiofrequency catheter ablation during surgery was performed as the first choice in our patient and did not result in any complications.

Table 1 is a brief review on some reported cases of GRAA and a systematic review.

**TABLE 1:** Brief review on some reported cases of GRAA and a systematic review

Author	Type of study	Clinical point
Kinsella A, et al^2^	Case report	Pre‐operative evaluation with echo and CMR
Zhang J et al.^3^	Systematic review	Analysis of 153 reported cases
Harder EE, et al.^4^	Case series	Neonatal manifestation with AT, fetal diagnosis with surgical repair at 4 months of age, fetal diagnosis with surgery at 15 months of age
Bachani N, et al.^5^	Case report	incessant atrial tachycardia and giant RA appendage aneurysm (surgical resection and MAZE)
Kobza R et al.^6^	Case report	RAA diverticulum and atrial flutter
Sivakumaran L et al.^7^	Case report	Evaluation of the role of CMR
Jonavicius K et al.^8^	Case report	A 16‐month‐old child with LVEF=30%, AT/AF
Binka E et al ^9^	Case report	Fetal Right Atrial Aneurysm and Aortic Coarctation with Left Ventricular Dysfunction
Narain VS et al.^10^	Case report	Right heart failure as the first presentation
Martín Talavera M et al.^12^	Case report	Prenatal diagnosis/surgery at 18 months+
Li H‐P et al.^15^	Two case reports	Prophylactic warfarin in one case/Administration of anticoagulation and amiodarone for atrial flutter
Melo H et al.^18^	Two case reports	A 14‐month‐old case with SVT and a 14‐year‐old male, both underwent surgical resection

Abbreviation: AF, atrial fibrillation; AT, atrial tachycardia; CMR, cardiac magnetic resonance; GRAA, giant right atrial aneurysm; RA, right atrium; SVT, supraventricular tachycardia.

## 6 CONCLUSIONS

As a rare congenital anomaly, a congenital right atrial aneurysm can be diagnosed incidentally or present with various symptoms, such as supraventricular arrhythmias, intra‐cardiac thrombus formation, heart failure, and even tamponade.^8^ Small asymptomatic aneurysms can be treated conservatively. Surgery is reserved for large symptomatic aneurysms. Open heart aneurysmectomy using cardiopulmonary bypass can be performed as a safe approach to repair the aneurysmal sac and other possible associated heart defects present. Early surgical resection to prevent atrial and electrophysiological remodeling may be considered.

## CONFLICT OF INTEREST

None declared.

## AUTHOR CONTRIBUTIONS

Seyed Mohsen Mirhosseini involved in data gathering and writing the text. Mahmood Beheshti Monfared and Mohammad KHani involved in data gathering. Sepideh Jafari Naeini involved in editing the text and writing.

## ETHICAL STATEMENT

Written informed consent was obtained from the patient who participated in this study. This case report did not receive any funding. The authors have access to all source data for this case report.

## CONSENT

Written informed consent was obtained from the patient to publish this report in accordance with the journal's patient consent policy.

## Supporting information

Video S1

Video S2

## Data Availability

The data that support the findings of this study are available on request from the corresponding author. The data are not publicly available due to privacy or ethics restrictions.
